# Diversity Among Cyanobacterial Photosystem I Oligomers

**DOI:** 10.3389/fmicb.2021.781826

**Published:** 2022-02-24

**Authors:** Ming Chen, Xuan Liu, Yujie He, Ningning Li, Jun He, Ying Zhang

**Affiliations:** ^1^The Seventh Affiliated Hospital, Sun Yat-sen University, Shenzhen, China; ^2^Center for Cell Fate and Lineage (CCLA), Bioland Laboratory (Guangzhou Regenerative Medicine and Health Guangdong Laboratory), Guangzhou, China; ^3^China–UK Institute for Frontier Science, Shenzhen, China; ^4^Tomas Lindahl Nobel Laureate Laboratory, The Seventh Affiliated Hospital, Sun Yat-sen University, Shenzhen, China; ^5^Center for Cell Lineage and Development, Guangzhou Institutes of Biomedicine and Health, Chinese Academy of Sciences, Guangzhou, China

**Keywords:** photosystem I, structure, cyanobacteria (blue-green algae), oligomers states, supercomplexes organization

## Abstract

Unraveling the oligomeric states of the photosystem I complex is essential to understanding the evolution and native mechanisms of photosynthesis. The molecular composition and functions of this complex are highly conserved among cyanobacteria, algae, and plants; however, its structure varies considerably between species. In cyanobacteria, the photosystem I complex is a trimer in most species, but monomer, dimer and tetramer arrangements with full physiological function have recently been characterized. Higher order oligomers have also been identified in some heterocyst-forming cyanobacteria and their close unicellular relatives. Given technological progress in cryo-electron microscope single particle technology, structures of PSI dimers, tetramers and some heterogeneous supercomplexes have been resolved into near atomic resolution. Recent developments in photosystem I oligomer studies have largely enriched theories on the structure and function of these photosystems.

## Introduction

Photosystem I (PSI) is a light-driven membrane protein complex that transfers electrons from plastocyanin/cytochrome C to ferredoxin/flavodoxin in oxygenic photosynthetic organisms (Amunts and Nelson, 2009). In cyanobacterial species, the PSI consists of 11 or 12 proteins coordinating approximately 127 cofactors that are responsible for the initial steps of light energy-induced electron transfer during photosynthesis. The current detailed understanding of the 3D structure of PSI originated from a study in cyanobacteria, in which a structure with 2.5Å resolution was established using X-ray crystallography (Jordan et al., 2001). Novel structural features, such as molecular interfaces, subcomplex organization, cofactors, pigment binding and functional relatives have been recently identified using cryo-electron microscope (EM) single particle analysis in oxygenic organisms (Pan et al., 2018, 2021; Suga et al., 2019; Zheng et al., 2019).

The native assembly of the PSI complex is of broad scientific interest in photosynthesis research owing to its considerable variability. The predominant state of the PSI complex is a trimer in most cyanobacterial species (Jordan et al., 2001; Malavath et al., 2018; Hamaguchi et al., 2021), whereas monomer (El-Mohsnawy et al., 2010), dimer, tetramer (Watanabe et al., 2010; Li et al., 2014; Zheng et al., 2019) and some undefined higher order oligomers (Watanabe et al., 2014; Li et al., 2019) have been detected in some subclasses. Unraveling the oligomeric diversity among PSI complexes is essential to understanding photosynthesis and the evolution of photosystems. For instance, in *Thermosynechococcus elongatus*, PSI trimers can absorb more photons than monomers under low light intensities owing to its higher optical cross-section (Baker et al., 2014). PSI dimers are regarded as an evolutionary intermediate between the trimers in cyanobacteria and monomers in eukaryotic photosynthetic organisms. This review discusses the implications of structural and oligomeric diversity among cyanobacterial PSI supercomplexes.

## Photosystem I Subunits

**FIGURE 1:**
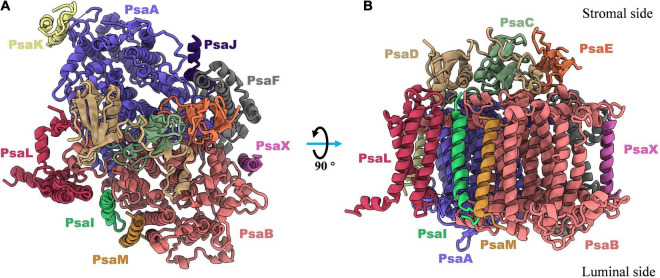
Top **(A)** and side **(B)** views of subunits composition in monomeric photosystem I complex from thermophilic cyanobacterium *Thermosynechococcus elongatus* (PDB: 1JB0). There are nine transmembrane proteins (PsaA, PsaB, PsaF, PsaJ, PsaK, PsaL, PsaM, PsaI, and PsaX) in which most of cofactors are non-charged bonded to perform light harvesting, light-to-electron energy conversion, and excitation transport. And three out membrane proteins (PsaC, PsaD, and PsaE) are located in stromal side and responsible for the electron transfer from PSI complex to the soluble acceptors.

The subunit composition of PSI complexes is highly conserved between cyanobacteria and plants. The structural and functional details of PSI proteins have previously been discussed in depth (Xu et al., 2001; Grotjohann and Fromme, 2005; Amunts and Nelson, 2009), and in this section of the review, a brief introduction to this topic is given. As shown in Figure 1, twelve subunits have been identified in cyanobacteria, nine of which (PsaA, PsaB, PsaF, PsaJ, PsaI, PsaK, PsaL, PsaM, and PsaX) are transmembrane proteins, and three of which (PsaC, PsaD and PsaE) are membrane extrinsic proteins, located on the stromal side of the PSI complex (Jordan et al., 2001).

PsaA, PsaB, and PsaC coordinate the major pigments for light reaction in photosynthesis, with central roles in light energy absorption and conversion; these proteins therefore comprise the core of the PSI complex (Nechushtai et al., 1983). PsaA and PsaB are transmembrane proteins with eleven transmembrane helices each. They share homology with each other and form a heterodimer in the redox center of PSI. These two subunits play essential roles in both light harvesting and electron transfer (Chitnis, 1996; Jordan et al., 2001). The main cofactors in the electron transfer chain (ETC)—P700, A, A0, A1 and Fx—are surrounded by the five C-terminal α-helices of the PsaA/PsaB subunits. These ETC cofactors are surrounded by the core antenna chlorophylls, also coordinated by PsaA/PsaB. The structure shows that most of chlorophyll A molecules in the reaction center bind with subunits PsaA and PsaB hydrophobically (Jordan et al., 2001). PsaC is an 8.8 KDa ferredoxin-like protein; together with PsaA and PsaB, this protein comprises the central core of PSI complex, harboring the ETC. Using biochemical reconstitution experiments, it has been shown that the PsaC polypeptide binds two [Fe_4_S_4_] clusters, F_*A*_ and F_*B*_, which are the terminal electron acceptors in PSI complex (Vassiliev et al., 1998). Interruption of the *psaC* gene in the filamentous cyanobacteria *Anabaena* sp. PCC 29413 led to the complete loss of the PsaC protein and its associated cofactors F_*A*_ and F_*B*_ and thus resulted in the absence of PSI-mediated electron transfer from P700 to ferredoxin (Mannan et al., 1991). PsaC is thought to mediate terminal electron transfer in the reaction center of the PSI complex (Klukas et al., 1999). Interestingly, a recent study in cyanobacteria revealed that PsaC interacts with ferredoxin in a similar way to a multi-protein complex-NDH-1L, which accepts electrons from ferredoxin (Zhang et al., 2020). This supports a role for PsaC as a core protein that mediates electron transfer from the reaction center of PSI to the outer electron acceptor chains.

PsaD and PsaE are peripheral proteins on the reducing side of PSI and are both involved in electron transfer (Yu et al., 1993; Chitnis et al., 1996). In biochemical studies in cyanobacteria, it has been demonstrated that PsaD provides a ferredoxin docking site, PsaE is also involved in ferredoxin binding to the PSI complex (Yu et al., 1993; Xu et al., 1994; Klukas et al., 1999). A high-resolution structure of the plastocyanin (Pc)–photosystem I (PSI)–ferredoxin oxidoreductase (Fd) supercomplex was recently resolved in the pea plant *Pisum sativum*, which clearly revealed a model for Fd–PSI binding (Caspy et al., 2020). From the structure, it has been concluded that the positively-charged amino acids in PsaD and PsaE composite a part of electrostatic interactions for the binding of ferredoxin to PSI complex. PsaF is an integral membrane protein with a molecular weight of 15.6 kDa. In green algae, PsaF is proposed to be a docking site for soluble electron donors, while in cyanobacteria, deletion of PsaF has no effect on photoautotrophic growth, as this protein is not essential for docking and electron transfer (Chitnis et al., 1991; Fischer et al., 1999). Structural studies revealed that a positively charged patch composed of Lys93, Lys96 and Lys100 in PsaF may be involved in correctly orienting Pc, aligning Cu^2+^ directly over P700 (Jordan et al., 2001). PsaI, PsaJ, PsaM, and PsaX are small transmembrane proteins in the cyanobacterial PSI complex, which stabilize the whole complex by interacting with the core subunits (Xu et al., 1995, 2001; Chitnis, 1996; Naithani et al., 2000).

**FIGURE 2:**
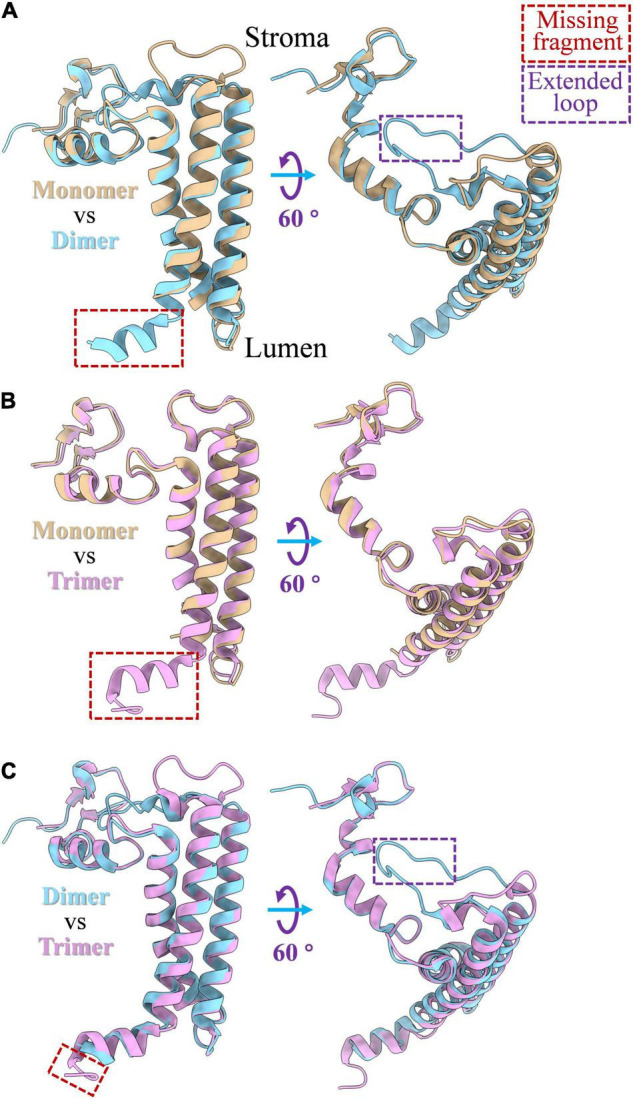
PsaL structure comparison among monomeric, dimeric and trimeric states of PSI complex. **(A)** Structure differences of PsaL between PSI monomer and dimer; **(B)** structure differences between of PsaL between PSI monomer and trimer; **(C)** structure differences of PsaL between PSI dimer and trimer. The losing fragments and extended loop regions were marked by red and purple rectangles, respectively. PSI monomer (PDB code: 6LU1) and trimer (PDB code: 1JB0) from *Thermosynechococcus elongatus* and PSI tetramer (PDB code: 6K61) from *Anabaena* sp. PCC 7120 were source molecules from which PsaL structures were extracted.

PsaL is a hydrophobic protein that is critical for the oligomerization of the PSI complex. The deletion of PsaL results in disassembly of PSI trimer in *Synechocystis* sp. PCC 6803 (Chitnis et al., 1993). The structure of PsaL subunits varies a little between monomeric, dimeric and trimeric states of the PSI; detailed structure differences are shown in Figure 2. An α-helix fragment at the luminal side (Figure 2B), which is involved in PSI trimer connections in *Thermosynechococcus elongatus*, was found to be missing when the PSI complex was biochemically monomerized (Jordan et al., 2001; Çoruh et al., 2021). Sequence alignments showed that the region involved in oligomerization is comprised of a 16 amino acid peptide, from Leu140 to Asn155 (Supplementary Figure 1B). In dimeric PSI, a similar α-helix fragment (Figure 2A) can be detected in the C-terminal of PsaL both from structural (Figure 2A) and sequence (Supplementary Figure 1A) data. As shown in Figure 2C, one additional structural variation in PsaL is that the dimer has a shorter C-terminal than the trimer by four amino acids (Supplementary Figure 1C). This may prevent the trimeric complex from forming, thus stabilizing the dimer (Jordan et al., 2001; Kato et al., 2019; Zheng et al., 2019; Chen et al., 2020). Interestingly, an extended loop is present in the dimer (Figures 2A,C); sequence alignments revealed that the loop is composed of six amino acids (Supplementary Figures 1A,C). The function of this loop in the oligomerization of the PSI dimer is currently unclear.

## Monomers

For decades, the PSI trimer was recognized as the only form with full physiological function in the model species of cyanobacteria (Boekema et al., 1987; Ford and Holzenburg, 1988; Kruip et al., 1994). The theory was challenged by El-Mohsnawy and coworkers, who isolated an intact monomeric form in which all subunits and photochemical activities of trimer were preserved (El-Mohsnawy et al., 2010). The monomeric state is recognized as a structural and functional intermediate during PSI trimer biogenesis and assembly. More importantly, the dynamic equilibrium between monomers and trimers may be critical for cyanobacteria to adapt to changing ecological environments (El-Mohsnawy et al., 2010; Yang et al., 2015). Most recently, high resolution structures of monomeric PSI complexes were resolved in *Synechocystis* sp. PCC 6803 and *Thermosynechococcus elongatus* using X-ray crystallography and cryo-EM single particle analysis, respectively (Netzer-El et al., 2019; Çoruh et al., 2021). Both structures clearly revealed that the subunit composition of monomers are highly identical to trimeric PSI. In *Thermosynechococcus elongatus*, the major difference was in the cofactors, in which 14 chlorophyll molecules detected in trimer were not be able to be built in atomic model of monomer. However, very few chlorophylls (aC-M1601 of PsaM and aC-K1401 of PsaK) at the monomer–monomer interface in the PSI trimer were found to be missing from the structure. This is an unexpected feature, as monomer isolation and purification preparation can result in the disassociation of cofactors located near the interface in the trimer.

Surprisingly, the chlorophylls that were located in the PsaL trimerization domain were present in the monomeric structure in *Thermosynechococcus elongatus*. However, through resolving the PSI monomeric structure from a PsaL modified strain of *Synechocystis* sp. PCC6803, Çoruh and coworkers found that disruption of a short helix in the PsaL C-terminus region, which stabilizes trimerization, can result in monomerization and loss of PsaL-associated pigments (Çoruh et al., 2021). One of the most important pieces of information to be drawn from studies of PSI monomers is the structural basis for the loss of red chlorophylls in cyanobacteria, which have essential functions for energy absorption and excitation of the complex under low light conditions. In *Thermosynechococcus elongatus*, between nine and eleven chlorophylls, with a red shift spectroscopic absorption of over 700 nm (long wavelength), were detected in each monomer unit of the PSI trimer (Pålsson et al., 1998; Jordan et al., 2001). Spectroscopic and structural characterization of both monomeric and trimeric PSI complexes strongly indicate that chlorophyll B1233 and PsaX-adjacent chlorophylls are long-wavelength chlorophylls (Skandary et al., 2015; Toporik et al., 2020).

## Dimers

**FIGURE 3:**
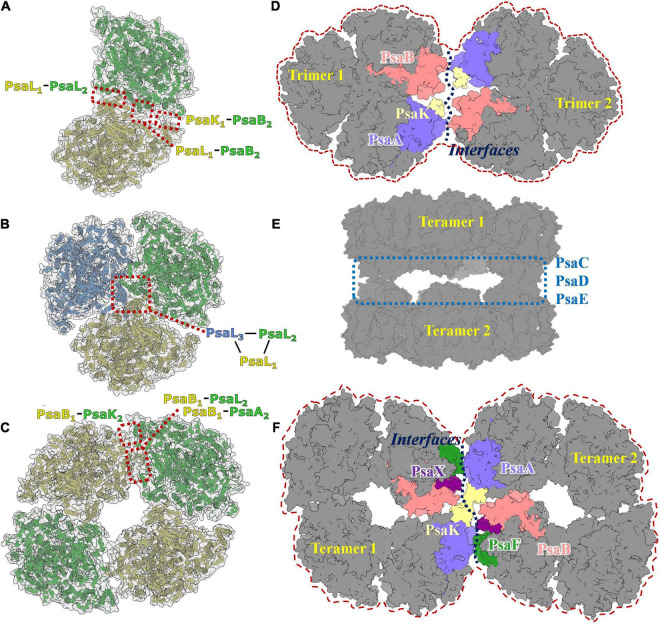
Photosystem I diversities in cyanobacteria. **(A)** Model of PSI dimer in *Anabaena* sp. PCC 7120 (PDB code: 6K61). **(B)** Model of PSI trimer in *Thermosynechococcus elongatus* (PDB code:1JB0). **(C)** Model of PSI tetramer in *Anabaena* sp. PCC 7120 (PDB code: 6TCL). **(D)** Predicted 3D map of PSI hexamer architecture. The interface involves subunits of PsaA, PsaB, Psak, PsaI, PsaM, and PsaL were colored and labeled separately. **(E)** Cryo-EM single particle density map of two face-to-face associated PSI tetramer complex. **(F)** Predicted 3D map of PSI octamer architecture. It should be realized that the models revealed in Figures 3A–C,E are experimental resolved and reported in the literature, while models revealed in Figures 3D,F are speculated according to the acknowledged structure basis of PSI dimer, trimer and tetramer.

The PSI dimer is one of the predominant oligomeric states in heterocystous cyanobacteria, and the high-resolution structure of the PSI dimer has been resolved in *Anabaena* sp. PCC 7120 (Zheng et al., 2019; Chen et al., 2020). As shown in Figure 3A, the dimer is composed of two monomers connected *via* specific interfaces. The interfaces are composed of subunits PsaL and PsaK from monomer1, PsaL and PsaB from monomer2 (Zheng et al., 2019). In contrast to the PSI trimer, four residues in PsaL at the C-terminus are missing from the luminal side in *Anabaena* sp. PCC 7120, resulting in a conformational change and slight movement of PsaL protein out from the C-terminal domain at the interface. Thus, it has thus been suggested that PSI dimerization occurs mainly *via* the stromal helices of PsaL (Chen et al., 2020). As a result, the connection between the two adjacent monomers is weaker compared with the trimeric state. Structurally, the distance between two chlorophylls that form the energetic coupling of the two monomers is greater, which suggests that the adjacent monomers in the dimer function more independently than in the trimer, owing to looser connections and longer distances.

Even though the PSI dimer has been intensively characterized in cyanobacteria, it was first observed in spinach, and was suggested to be an artificial aggregation of monomers resulting from membrane solubilization (van Roon et al., 2000). Large amounts of PSI–light harvesting complex I (LHCI) dimers were observed in the stromal lamellae of dark-adapted spinach thylakoids using atomic force microscopy (AFM) technology (Wood et al., 2018). A high-resolution structure (2.97Å) of the chloroplast PSI–LHCI dimeric supercomplex has been resolved very recently (Naschberger et al., 2021). It was clearly shown in this structure that the interface in the PSI–LHCI dimer is composed of the LHC9 protein and its associated bound lipids and pigments, which is a distinct molecular link for PSI oligomerization from that of cyanobacteria. The physiological function of the PSI–LHCI dimer complex is not well defined in higher plants; however, the dimeric arrangement seems to be dark-responsive (Wood et al., 2018).

## Trimers

In most species of cyanobacteria, PSI complexes assemble as a cloverleaf-shaped trimer with a diameter of 220Å (Figure 3B) in the native thylakoid membrane, in which the linker peptide is PsaL. Boekema and coworkers (Boekema et al., 1987) obtained the very first evidence for trimerization of the PSI reaction center from a thermophilic cyanobacterial species. The PsaL subunit plays a pivotal role in forming the interface between PsaL are the key force to link monomers to form trimer were detected on experimental deletion of the PsaL subunits. High resolution (2.5Å) structure (PDB entry: 1JB0) of the PSI trimer has been obtained from *Thermosynechococcus elongatus* using X-ray crystallography (Jordan et al., 2001), in which details of the complex architecture, molecular locations, compositions, and interactions were presented clearly for the first time. Nine transmembrane subunits (PsaA, PsaB, PsaF, PsaI, PsaJ, PsaK, PsaL, PsaM, and PsaX) and three stromal attached subunits (PsaC, PsaD, and PsaE) were present in this structure (Figure 1). The positions of all subunits were revealed in the core structure, which were consistent with biochemical and biophysical evidences (Boekema et al., 1987; Li et al., 1991; Chitnis et al., 1993; Vassiliev et al., 1998; Fischer et al., 1999; Klukas et al., 1999).

Unlike the PSI dimer, the interface between monomeric units in the PSI trimer complex mainly consists of the PsaL subunit. The cofactor framework for light energy conversion and transfer was also drawn clearly from this model. In 2018, Malavath and coworkers resolved a structure with 2.5Å resolution (PDB entry: 5OY0) for the PSI trimer from the mesophilic cyanobacterium *Synechocystis* sp. PCC 6803 using X-ray crystallography (Malavath et al., 2018; Netzer-El et al., 2019). The molecular composition, positions, and interfaces in this structure were mostly identical to that of *Thermosynechococcus elongatus*; however, more detailed information could be derived from this model. There were found to be several novel structural features compared with the PSI trimer from *Thermosynechococcus elongatus*, including the absence of nine chlorophylls in the trimeric model, the replacement of PsaX with chlorophyll F1302, and several new carotenoids and lipids were determined for the first time from this structure.

In recent years, new PSI complex structures have been resolved in some cyanobacterial species that utilize far-red light (Gisriel et al., 2020; Hamaguchi et al., 2021) and are adapted to extremely high light intensities (Dobson et al., 2021). The assembled PSI trimers in these species has a similar shape to the trimers from *Thermosynechococcus elongatus* and *Synechocystis* sp. PCC 6803. Further, PSI trimers has been established as the most predominant oligomeric state in cyanobacteria *via* the structural analyses described here.

## Tetramers

Tetrameric PSI complexes are novel supercomplexes that were first detected in *Anabaena* and *Cyanophora paradoxa* using blue native polyacrylamide gel electrophoresis (BN-PAGE); (Watanabe et al., 2010). Molecular weight and sodium dodecylsulfate (SDS)-PAGE-based molecular composition assays have strongly suggested that the larger detected band on the BN-PAGE is a tetrameric PSI complex. The existence of the PSI tetramer was demonstrated in the heterocystous cyanobacterium *Anabaena* sp. PCC 7120 using single particle transmission electron microscopy analysis (TEM; Watanabe et al., 2014). The first cryo-EM structure of the PSI tetramer was obtained by Semchonok and coworkers from the thermophilic cyanobacterium *Chroococcidiopsis* sp. TS-821 (Semchonok et al., 2016). This 11.5Å resolution revealed that the PSI tetramer is a pseudotetramer, consisting of a dimer of dimers (Figure 3C). Two types of interfaces were presented in PSI oligomerization based on the electron density map, including one novel type. Interface features have been shown using rigid body-fitting for the monomers within the crystal structure of the PSI trimer from *Thermosynechococcus elongatus*. The subunits PsaA, PsaB, PsaI, PsaL, and PsaM are involved in the interface I, while PsaA, PsaB, and PsaL are the mainly proteins in interface II. Recently, high-resolution structures of PSI tetramers have been resolved from *Anabaena* sp. PCC 7120 using cryo-EM single particle analysis (Kato et al., 2019; Zheng et al., 2019; Chen et al., 2020). The monomeric models from *Thermosynechococcus elongatus* and *Synechocystis* sp. PCC 6803 were used as initial models to build and refine atomic models of the PSI tetramer. No significant conformational variations in the interacting protein subunits of monomer complexes were detected among these models. Interface I is present in PsaL_1_–PsaB_2_, PsaL_1_–PsaL_2_, and PsaK_1_–PsaB_2_ interactions; this is similar to the interfaces in the trimeric PSI from *Thermosynechococcus elongatus*. Unlike interface I, the interface II occurs in the PsaB_1_–PsaA_2_, PsaB_1_-PsaL_2_, and PsaB_1_-PsaA_2_ interactions. A tight hydrophobic interaction between adjacent PsaL subunits is the primary connection in the formation of dimer units, as was observed in trimeric complexes. PsaL subunits also have a vital role in the assembly of tetramers. Research revealed two additional polar contact sites in interface I; one between Arg85 of PsaK from one monomer and Ser214 of PsaB’ from the adjacent monomer, and one between the two stromal side residues of PsaL and the two C-terminal residues of the adjacent subunit PsaL’ (Zheng et al., 2019).

To explore why monomers form dimers instead of trimers given the structural similarities in interface I, Kato and coworkers superimposed a monomeric unit of the PSI tetramer onto the corresponding site in the PSI trimer (Kato et al., 2019). Several steric hindrances were found to prevent formation of the trimeric core, which were contributed mainly by the N-terminal, middle and C-terminal regions of the PsaL subunit. Among these residues, Thr58, Phe60, Arg61, Asn140, and Ser144 were found to be conserved in species in which the PSI tetramer was formed. Some interface variations were detected in the validated model of the PSI tetramer. For instance, a short luminal C-terminal residue in PsaL was found to be missing in *Anabaena* and this was reported to form a coordination site for Ca^2+^ ions that stabilize the trimer (Chen et al., 2020). One interesting finding is that a tilting was detected in both PsaI and PsaL subunits, introducing 5–6Å movements away from the interface that stabilized the oligomerization of monomers. This tilting ultimately resulted in a shift of the monomer and a loosening of PsaL-mediated monomer interactions that generate the internal connections within PSI dimer. Another significant structural variation is that the distance between chlorophylls forming exitonic couplings in the PSI trimer was increased from 17.0 to 20.7Å in the dimer, supporting the functional variation in PSI dimer where each monomer functions more independently than in the trimer (Chen et al., 2020).

## Higher Order Oligomers

Higher order oligomeric PSI complexes are rarely reported in cyanobacteria, algae, or green plants. Through negative staining single particle TEM analysis, Watanabe and coworkers averaged a projection map of shoulder-to-shoulder associated PSI tetramer (Watanabe et al., 2014). Further biochemical and biophysical studies are needed to evaluate whether this is a PSI octamer. In our previous work, a novel structure of a dimer of PSI tetramers was reconstructed and refined to resolution of 6–8Å (Chen et al., 2020). In this complex, two PSI tetramers are present with face-to-face stromal domains formed *via* direct interactions between PsaC, PsaD, and PsaE modules (Figure 3E). This indicates that such a complex is probably not physiologically relevant, and thus not a PSI octamer complex. In these studies, the major doubt for the existence of native higher order PSI complexes comes from a lack of biochemical evidence, such as the presence of PSI complex bands with molecular weight larger than that of the tetramer on native blue PAGE, and purification of the stable complex from cyanobacteria.

In a recent study, Li and coworkers identified a possible PSI hexamer in *Chroococcidiopsis* sp. PCC 6712 using BN-PAGE technology (Li et al., 2019). No high-resolution structure has been resolved for these supercomplexes so far; however, assembly models for PSI hexamer and octamer can still be hypothesized based on reported structural details of molecular interfaces in PSI dimers and tetramers, and on the negatively stained image of a possible PSI hexamer in *Chroococcidiopsis* (Li et al., 2019) and PSI octamer in *Anabaena* (Watanabe et al., 2014). As shown in Figures 3D,F, theoretical models of the PSI hexamer and octamer can be drawn based on the interfaces involved in dimer and tetramer formation between PsaA, PsaB, PsaF, PsaK, PsaL, PsaM, PsaI, and PsaX subunits (Zheng et al., 2019; Chen et al., 2020). Using newly developed isolation methods, we obtained a pure sample of PSI-like supercomplexes with molecular weights between 2000 and 2800 kDa. Structural and functional characterization of these supercomplexes are underway, from which further details of these complexes may be inferred.

An interesting study on the biogenesis of cyanobacterial thylakoid membranes was recently published (Huokko et al., 2021), in which it was shown that the assembly of photosynthetic membrane complexes respond dynamically to changing light strength. The assembly of PSI complexes in cyanobacterial cells are largely improved during a 5-day dark adaptation period. The photosynthetic complexes may be reorganized under such dynamic circumstances. Recent atomic force microscopy results clearly show that the photosynthetic complexes attach to each other to form megacomplexes with dynamic architectural reorganization of the native thylakoid membrane (Zhao et al., 2020). Such dynamic rearrangements in membrane complexes have been regarded as an important mechanism for living cells to adapt to variations in ecological environments, as in the phycobilisome (PBS)-iron stress-induced protein A (isiA)-NADH dehydrogenase-like complex type-1 (NDH-1) supercomplex (Zhang et al., 2010; Casella et al., 2017; MacGregor-Chatwin et al., 2017; Zhao et al., 2020). PSI tetramers have been presented as highly light-responsive complexes in *Chroococcidiopsis* sp. TS-821. PSI oligomer state equilibrium is considered as one means by which energetic metabolism is balanced in living cyanobacterial cells (Li et al., 2014). Taking the above into account, higher order PSI supercomplexes in native membranes may be structurally and functionally important for efficient energy metabolism and even survival in cyanobacteria under stress condition. All these studies have indicated that unknown oligomer states of PSI supercomplexes with specific physiological functions can be detected, especially in cells faced with variable ecological environments.

## Conclusion

Photosystem I is one of the most important multiprotein membrane complexes in oxygenic photosynthetic organisms. An understanding of homogenous and heterogenous PSI supercomplexes is required to understand the native mechanisms of photosynthesis. The diversity in PSI oligomeric states among cyanobacteria has garnered considerable interest recently, because resolving high resolution structure of these supercomplexes is now technically feasible. At least four different kinds of oligomer have been functionally and structurally demonstrated in cyanobacteria. Such diversity in oligomeric states is now acknowledged to be functionally important, helping cells to survive under changing ecological environments. Monomeric PSI exhibits a complete range of physiological functions in cyanobacteria compared with the previously-established range for the trimer. Meanwhile the trimer harvests light more efficiently under low light conditions, and the tetramer is more prevalent in high-light conditions. The tetramer is composed of a dimer of dimers; the dimer is now regarded as an evolutionary intermediate state between the trimer in cyanobacteria and the monomer in green algae and higher plants. Given the advantages of cryo-EM single particle structural analysis of supercomplexes, novel molecular features, including conformers, assembly models, interfaces, and even ligand binding were acknowledged to help unravel energy-trapping and electron transfer in PSI complexes. Furthermore, some newly higher order PSI oligomers have also been detected in cyanobacteria, algae and higher plants. This indicates that the assembly of native PSI complexes is more complicated than previously thought, and its characterization is key to understanding the role of these complexes in photosynthesis.

## Author Contributions

MC, XL, YH, and YZ wrote the manuscript with adding from NL, JH, and YZ supported the research with funding acquisition. All authors read and approved the manuscript.

## Conflict of Interest

The authors declare that the research was conducted in the absence of any commercial or financial relationships that could be construed as a potential conflict of interest.

## Publisher’s Note

All claims expressed in this article are solely those of the authors and do not necessarily represent those of their affiliated organizations, or those of the publisher, the editors and the reviewers. Any product that may be evaluated in this article, or claim that may be made by its manufacturer, is not guaranteed or endorsed by the publisher.
