# Supplementary material for: Diversity Among Cyanobacterial Photosystem I Oligomers
Source: Front Microbiol. 2022 Feb 24;12:781826. doi: 10.3389/fmicb.2021.781826 (PMC8908432; doi:10.3389/fmicb.2021.781826)
Supplement: Supplementary file 1 [file Data_Sheet_1.docx]

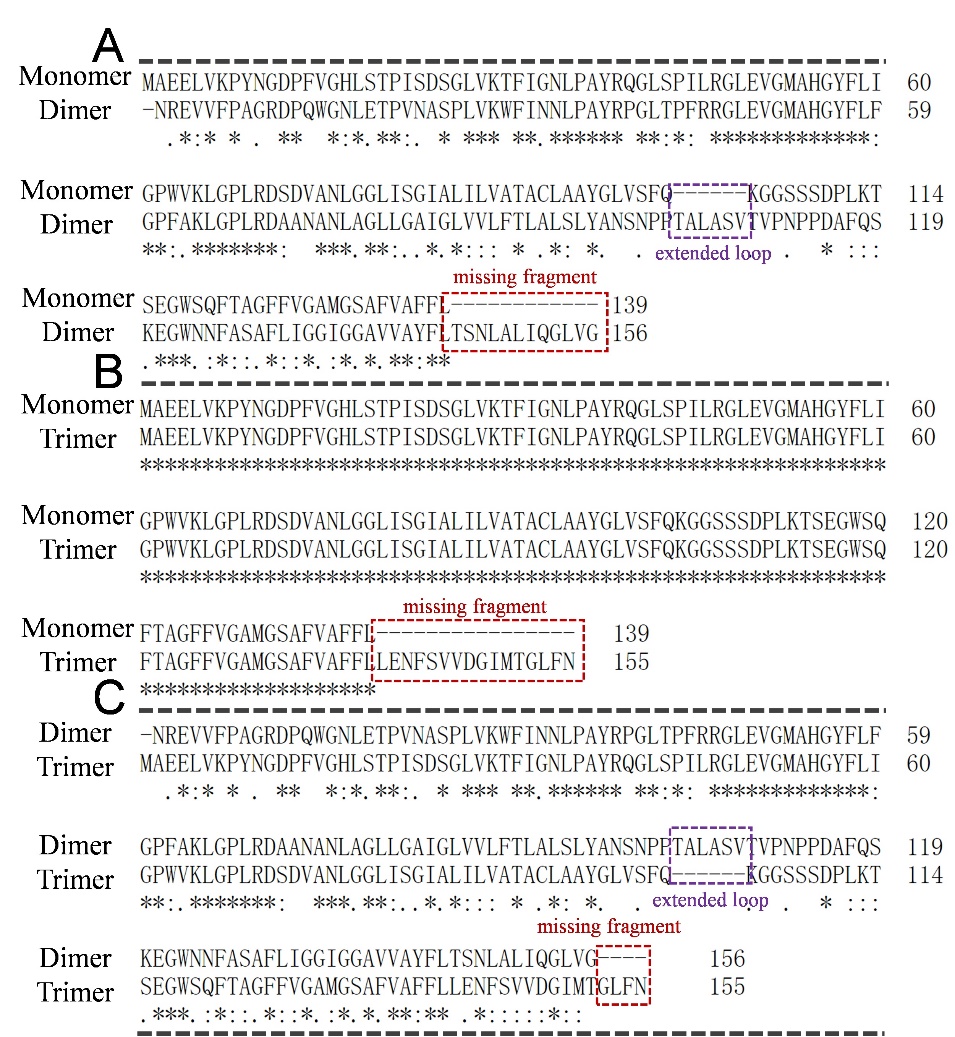


Supplementary figure 1-The PsaL sequence alignment of from PSI monomer and dimer (A), monomer and trimer (B), and dimer and trimer (C). The sequences were extracted from the structure of monomer (PDB code:6LU1), dimer (PDB code: 6K61) and trimer (PDB code: 1JB0) respectively from ChimeraX program. The alignment was calculated in web tool of Uniprot. The sequences for missing fragments and extended loop were indicated by the red and purple rectangles.
